# Psychological Reactions and Persistent Facial Pain following Enucleation

**DOI:** 10.1155/2014/232989

**Published:** 2014-04-23

**Authors:** Dominic Hegarty, Daniel Coakley, Ian Dooley

**Affiliations:** ^1^Department of Anesthesia & Pain Medicine, Cork University Hospital, Cork, Ireland; ^2^Department of Ophthalmology, Cork University Hospital, Cork, Ireland

## Abstract

*Background*. Enucleation is a psychologically and physically traumatic event associated with chronic pain. It would be desirable to better predict which patients will have pain after surgery. *Methods*. A cross-sectional postal questionnaire study of adults undergoing enucleation captured the demographic details, Pain Quality Assessment Scale (PQAS), Pain Catastrophizing Scale (PCS), and the Facial Pain Assessment questionnaire. Patients were classified as suffering from chronic pain if they reported a pain score of >1 out of 10 on the numerical pain score (NRS). *Results*. Seventeen of 60 adults participated in the study. 47% of patients reported chronic pain (mean pain score = 1.4 ± 0.7, *n* = 17); 25% experienced pain daily. No difference in age, surgical side, reason for surgery, or the duration of time since the surgery was noted. All patients had low PQAS scores and 50% of individuals with persistent pain were concerned about their facial appearance. There was no significant difference in the level of catastrophization noted in patients with or without pain or between the subgroups (rumination, magnification, or helplessness). *Conclusions*. Although persistent pain following enucleation affected a significant number of patients, the pain intensity was mild. Enucleation influenced the physical perception some individuals had of themselves.

## 1. Introduction

Enucleation is often the final step in a complicated series of eye disease treatments. It is indicated in the treatment of severe ocular trauma, ocular pain, and in the treatment of intraocular neoplasms. Ocular surgery has being shown to have a significant impact on the quality of life and function of affected individuals [1]. It is usually effective in resolving pain [2, 3], but not always [4, 5]. Persistent postsurgical pain (defined by the International Association of the Study of Pain as pain that develops after surgery and has been present for at least 3 months [6]) affects between 10% and 50% of patients after enucleation [7].

We sought to evaluate the prevalence and character of persistent pain (i.e., neuropathic or nonneuropathic pain) following enucleation and examine the association between persistent pain and biopsychosocial profile of these adults. Greater insight into the relationship between degree of pain and the psychological impact of the diagnosis may assist in determining the preferred management and help refine postoperative routines for future patients with enucleation.

## 2. Methods

### 2.1. Participants

A cross-sectional questionnaire study of all adults who had enucleation from 2005 to 2011 at our institution was performed. Individuals were identified using the hospital database code (ICD-10-AM) and Hospital In-Patient Enquiry Scheme. Medical records were then consulted to clarify the ophthalmic diagnosis and operative technique employed. A designated investigator (DC) then contacted the individuals by phone to invite them to participate in the study. Inclusion criteria were as follows: (i) greater than 18 years of age at the time of enucleation; (ii) English speaking; (iii) recovery of at least 12 months after surgery; (iv) no history of additional facial surgery or trigeminal neuralgia since the enucleation; and (v) consent to participate in the study. Ethical approval for this study was obtained from the Cork University Hospital Group Ethics Committee (reference number 232412).

A questionnaire pack designed for ease of completion was prepared and tested in advance of its distribution to study participants. It was anticipated that completion would take 15 minutes. A cover letter to clearly explain the nature of the study and instructions for each individual was provided. Individuals were asked whether they would prefer large font questionnaires. A self-addressed envelope was included for return of the questionnaire. Three weeks after mailing the questionnaires, a follow-up telephone call was made to those individuals who had failed to return the pack. An additional questionnaire was reposted if the original was mislaid or if telephone contact was not achieved after repeated attempts. Each patient received a maximum of one telephone reminder and one postal reminder. Any incomplete or ambiguous information in the returned questionnaires was clarified by telephone, when possible.

## 3. Assessments

### 3.1. Demographic Details

Demographic details were obtained from the hospital notes and updated to capture marital status, psychological profile, and employment records at the time of the study.

### 3.2. Pain Quality Assessment Scale (PQAS)

Patients were asked to rate the average pain intensity in their orbit/face over the past week on a 0–10 numerical rating scale (where 0 = “no pain” and 10 = “the most intense pain sensation imaginable”). This indicated the presence or absence of persistent pain following enucleation. Patients were divided into two groups, those without pain (pain score = 0) and those with pain (pain score > 1).

Clinical outcome was assessed using the Pain Quality Assessment Scale (PQAS) [8]. Each PQAS item assessed a different pain domain or quality. Patients were asked to rate the severity of each of 20 pain (quality and spatial) descriptor domains by using 0 to 10 numeric rating scales, in which 0 = “no pain” or “not (sensation/item)” and 10 = “the most (descriptor) pain sensation imaginable.” Fifteen of the items were used to create three PQAS scores that assessed paroxysmal, surface, and deep pain. These scales were validated using an initial exploratory factor analysis and subsequent confirmatory factor analysis. Both analyses demonstrated that the items selected clustered into the three domains in a consistent manner. The responses to the PQAS items were also analyzed individually.

### 3.3. Facial Pain Assessment

Information concerning facial appearance, distress related to perceived appearance, impaired occupational and/or social functioning, and frequency and character of facial pain was captured using a Facial Pain Assessment questionnaire presently being developed at Cork University Hospital. The assessment incorporated a facial drawing to capture the pattern of pain. A ptosis chart is a novel method that was used to measure a patient's perception of their facial image. The patient selected from 5 pairs of eye drawings with varying degrees of ptosis the drawing that most closely matched their appearance.

### 3.4. The Pain Catastrophizing Scale

The Pain Catastrophizing Scale (PCS) consists of 13 items rated on a 5-point scale ranging from 0 (not at all) to 4 (all the time). Participants were instructed to indicate the degree to which they had specific thoughts and feelings when experiencing pain. The measure assesses three dimensions of catastrophizing: rumination, magnification, and helplessness. The PCS has been validated for both clinical and nonclinical samples [9]. A total PCS score of 30 represents a clinically relevant level of catastrophizing and corresponds to the 75th percentile of the distribution of PCS scores in clinic samples of chronic pain patients.

### 3.5. Data Analysis

All data recorded in the questionnaires was transferred to a secure Excel database. Statistical analyses were carried out using SPSS 16.0 software for windows. Data was expressed as the mean ± standard deviation (SD). Descriptive analysis, Student's *t*-test, Pearson's Chi-square testing, and other appropriate analyses were performed. *P* < 0.05 was considered statistically significant.

## 4. Results

Hospital records identified 60 adults with enucleation between 2005 and 2011. All were subsequently contacted. 12 patients declined to participate, 14 could not be reached, and 6 had died in the interim. Twenty-eight patients agreed by phone to participate in the study, but 11 of them failed to reply. In all patients, seventeen patients (28.4% of the patients undergoing enucleation) completed the questionnaire pack. Eight of the 17 responders (47.1%) reported the presence of persistent facial or orbital pain since their surgery. Their mean pain intensity score was low, 1.4 ± 0.7 (where 0 is no pain and 10 is the worst pain). Twenty-five percent (2/8) of these individuals experienced pain on a daily basis. Patients with and without chronic pain did not differ in age, surgical side, reason for surgery, or the duration of time since the surgery (Table 1). Persistent pain was reported more often in males, although the difference was not statistically significant (Table 1).

### 4.1. Pain Quality Assessment Scale

Individuals with persistent pain consistently reported abnormal sensations in all domains with the exception of “cold” and “sensitivity” (Table 2). The frequency of pain experienced by the group was evaluated (Table 3). Post hoc analysis did not identify any difference between neuropathic and nonneuropathic pain assessment elements (mean PQAS score for patients with neuropathic pain 1.6 ± 0.6 versus those with nonneuropathic pain was 1.4 ± 0.8; *P* = 0.5).

### 4.2. Facial Pain Assessment Tool

As only 8 patients reported pain, patients who reported a ptosis score of 4 or 5 were compared to those with a ptosis score less than 4 (Table 4). There was no significant association between the perceived degree of ptosis and the presence or absence of pain (*χ* ^2^(1) = 0.9, *P* = 0.34 (odds ratio = 0.6)). 50% of individuals with persistent pain were concerned about their facial appearance and reported being “distressed” about it (Table 5).

### 4.3. Pain Catastrophizing Scale

The mean total pain catastrophizing score was low, less than 30, and no significant difference in the level of catastrophization was noted in patients with or without pain (Table 6).

## 5. Discussion

Almost half (47%) of the patients who responded reported mild to moderate pain persistent up to 4.3 years after their enucleation surgery. The character of the pain was both neuropathic and nonneuropathic in nature. Most patients did not require oral analgesics to manage the pain. No demographic, psychological, or anatomical associations were useful in predicting those at risk of developing persistent pain.

The development of persistent postsurgical pain (PPSP) in other surgical cohorts is well documented and ranges from 10 to 50% [10]. Our finding of PPSP in 47% of responding patients is similar to these reports. The causes of persistent pain after enucleation include nonoptimal fit of prosthesis, migration of the implant, inflammatory conditions, phantom pain, space-occupying lesions, painful microscopic amputation neuroma, and psychiatric disorders.

Phantom pain is thought to be related to changes in the cortical representation of body parts adjacent to the amputated area [11] and is a common finding after limb amputation, occurring in up to 78% of patients [12]. Despite a reported incidence of 22% following enucleation [13], little is known about this phenomenon; however, this could be attributed to the smaller cortical somatosensory representation of the eye, compared to that of the limbs.

Differentiation of the character of the pain as neuropathic or nonneuropathic pain (i.e., nociceptive or mixed features) [10] is important because it influences treatment and prognosis. Neuropathic pain presents as spontaneous pain, hyperalgesia, and fluctuations in pain sensitivity to stimuli. It is maladaptive and more likely to be found following surgery. The increased flow of pain signals after surgery sensitizes the neurons in the dorsal horn of the spinal cord and further amplifies the sensation of pain. This central sensitization can profoundly affect the experience of pain [14].

It is noteworthy that the pain catastrophizing scores of affected patients in our study were low. In general, individuals with chronic pain have a tendency to focus on and exaggerate the threat of painful stimuli and therefore tend to negatively evaluate their ability to manage their pain, subsequently leading to higher catastrophizing scores. In addition, catastrophizing is preferentially associated with emotional responses to pain in younger adults (<65 years) and with a higher pain intensity rating in older adults (>65 years) [15]. Our results mirror this pattern. The lower pain intensity was reflected in the low catastrophizing scores of this elderly cohort.

The loss of body parts can have distinct, but overlapping, psychological consequences. These include bodily changes—alterations in the way patients, their families, and others perceive their bodies—or changes of function—alterations in the activities and roles that they are able to carry out [16]. The Facial Pain Assessment tool demonstrated limited ability to identify patients with chronic pain. About half of the individuals with pain were concerned about their facial appearance following enucleation, causing them distress or impaired function. This finding merits further study.

Body image, as defined by McCrea [17], is “the subjective evaluation of one's own body and the feelings and attitudes attached to this evaluation.” Self-figure drawing is a recognized technique in psychiatry to externally represent a patient's own image [18]. Osman et al. [9] used self-figure drawings to examine the relationship between drawing size and self-esteem in students. We are developing a novel-screening tool, the relative prosthetic eye size chart, to assess a patient's self-image following enucleation. Five of eight patients with eye pain identified themselves as having abnormally large eyes, compared to 3 of 9 patients without eye pain. Although our study failed to demonstrate statistical significance (*P* = 0.34 (Table 4)), the relative prosthetic eye size chart does show promise in identifying patients with adverse body image. Further studies with larger numbers of patients are needed to validate its use.

This study was designed as a self-reporting postal questionnaire; therefore, we cannot remark on the potential cause of pain in each patient. Pain intensity greater than mild levels (i.e., >3/10) is usually investigated. The low pain levels observed in our patients did not trigger further investigations.

A significant limitation of our study was the small sample size. Several attempts were made to obtain a response. Assael and Keon asserted that while sampling error makes a minimal contribution to total survey error, nonsampling error, the error that includes a nonresponse component, is a major contributor [19].

Nonresponse can be “technical” in nature, for example, a wrong address, lack of interest in the study, or a negative attitude to the health care system [1, 8]. Mayer and Pratt [20] found that the level of involvement and interest in the survey topic had an effect on nonresponse rates, while van Kenhove et al. found that topic involvement was not significantly different for respondents and nonrespondents [21].

Patients with health problems may be more likely to respond to symptom-orientated questionnaires than those without problems [8]. This finding was contradicted by other studies showing that persons feeling well were more likely to respond [9, 19]. Implications of nonresponse in our study include concerns about the number of nonrespondents and the possibility of bias. The usefulness of findings from studies with low response rates is an area of concern [22].

## 6. Conclusions

Although persistent pain following enucleation affected a significant number of patients, the pain intensity was mild. Enucleation influenced the physical perception some individuals had of themselves. The impact on quality of life requires further exploration. Larger numbers of patients are needed to evaluate these preliminary findings.

## Figures and Tables

**Table 1:** Patient demographics.

	No pain (*n* = 9)	Persistent pain (*n* = 8)	*P* value
Age			
(Mean ± SD, years) Range (years)	60.5 ± 18.2 (41−89)	60.7 ± 18.1 (23−81)	ns
Gender			
(Female/male)	6/3	2/6	*χ* ^2^(1) = 4.0, *P* < 0.5
Time since surgery (years)	3.4 ± 2.1	4.3 ± 2.7	
Surgical side			
Left	5	3	*χ* ^2^(1) = 0.56, *P* = 0.45, OR = 0.75
Right	4	5	
Reason for surgery			
Trauma	3	0	n.s
Cancer	2	2	
Infection	2	2	
Other	2	4	
Number of attendance times to GP in the last 12 months			
0−4	6	7	*χ* ^2^(1) = 1.01, *P* = 0.3
>5	3	1	

Where mean ± standard deviation (SD); *χ* ^2^ = chi-square test; OR = odds ratio.

**Table 2:** Pain Quality Assessment Scale (PQAS) for individuals with persistent postsurgical pain after enucleation (*n* = 8, *nonneuropathic pain elements).

PQAS	Mean (SD)
(1) Intense	1.3 (2.1)
(2) Sharp	2.2 (2.4)
(3) Hot	0.75 (1.4)
(4) Dull	1.6 (2.6)
(5) Cold	0
(6) Sensitive	0
(7) Tender*	0.5 (1.4)
(8) Itchy	1.8 (2.4)
(9) Shooting*	2.8 (3.9)
(10) Numb*	1.75 (3.2)
(11) Electrical*	0.75 (2.1)
(12) Tingling*	1.87 (2.7)
(13) Cramping*	0.87 (1.4)
(14) Radiating*	1.1 (2.2)
(15) Throbbing*	1.3 (2.6)
(16) Aching*	2.0 (2.8)
(17) Heavy*	1.87 (2.4)
(18) Unpleasant	2.1 (2.5)
(19) Deep	1.75 (2.5)
(20) Surface	2.1 (3.0)

**Table 3:** Incidence of persistent postsurgical pain following enucleation.

	Never	Monthly	Weekly	Daily
How often do you experience pain? (*n* = 17)	9	4	2	2
% total	53%	23.5%	11.75%	11.75%

**Table 4:** Ptosis and appearance score in individuals with enucleation.

Ptosis grade	No pain	Persistent pain	*P* value
4 or 5	3	5	*χ* ^2^(1) = 0.9, *P* = 0.34 (odds ratio = 0.6)
3 or less	6	3	

**Table 5:** Concern with facial appearance in patients with and without persistent pain following enucleation.

Individuals with persistent pain	Never	Seldom (less than half the time)	Over half of the time	All of the time
Concerned about facial appearance	4	1	2	1
Appearance causes distress or impaired functioning	4	2	2	0

**Table 6:** Pain Catastrophizing Scale (PCS) scores of patients with and without persistent pain after enucleation (mean (SD), Student's *t*-test).

PCS total score	No pain	Persistent pain	*P* (*t*-test)
Total score	6.8 (15.9)	3.6 (6.8)	0.29
Rumination (4 elements)	1.6 (4.0)	1.4 (2.6)	0.43
Magnification (3 elements)	1.5 (3.1)	1.0 (1.9)	0.33
Helplessness (6 elements)	3.2 (7.4)	1.2 (2.3)	0.24
